# Phase-change like process through bond switching in distorted and resonantly bonded crystal

**DOI:** 10.1038/s41598-019-49270-2

**Published:** 2019-09-06

**Authors:** Won Jun Yang, Hanjin Park, Da Sol Kim, Taewoo Ha, Seung Jong Park, Min Ahn, Jae Hoon Kim, Young-Kyun Kwon, Mann-Ho Cho

**Affiliations:** 1 Department of Physics and Applied Physics, Yonsei University, Seoul, 03722 Republic of Korea; 2 Department of Physics and Research Institute for Basic Sciences, Kyung Hee University, Seoul, 02447 Republic of Korea; 3 Present Address: Center for Integrated Nanostructure Physics, Institute for Basic Science (IBS), Sungkyunkwan University (SKKU), Suwon, 16419 Republic of Korea

**Keywords:** Electronic properties and materials, Electronic devices, Information storage

## Abstract

Although some methods to improve phase-change memory efficiency have been proposed, an effective experimental approach to induce a phase-change like process without external heat energy has not yet been reported. Herein we have shown that GeTe is a prototype phase-change material, which can exhibit a non-thermal phase-change-like process under uniaxial stress. Due to its structural characteristics like directional structural instability and resonance bonding under 1% uniaxial stress, we observed that bond switching in the GeTe film between short and long bonds is possible. Due to this phase change, GeTe displays the same phase-change as crystal layer rotation. Crystal layer rotation has not been observed in the conventional phase change process using intermediate states, but it is related to the structural characteristics required for maintaining local coordination. Moreover, since the resonance bonding characteristics are effectively turned off upon applying uniaxial stress, the high-frequency dielectric constant can be significantly decreased. Our results also show that the most significant process in the non-thermal phase transition of phase-change materials is the modulation of the lattice relaxation process after the initial perturbation, rather than the method inducing the perturbation itself. Finally, these consequences suggest that a new type of phase-change memory is possible through changes in the optical properties under stress.

## Introduction

A group of phase-change materials in the pseudobinary GeTe-Sb_2_Te_3_ tie-line (GST) has been widely used in memory devices^1,2^. Another group of phase-change materials, namely, Ag-In-Sb-Te alloys (AIST), which are based on different phase-change dynamics has also been extensively studied for various applications^3–5^. In order to apply the materials in storage device development, the possibility of information storage using contrast difference in optical and electrical changes arising from the thermally-induced phase transition between a partially covalently-bonded “amorphous phase” and the resonantly-bonded “crystalline phase” with inherent distortions, has been explored^6–9^. With excellent properties such as desired scalability, endurance, and on-off ratio, phase-change memory has been commercialized and shipped as storage class memory rather than as universal memory owing to its limited switching speed and high power consumption. To overcome this switching efficiency problem, studies to develop an energy-efficient channel have been actively conducted^10–12^. However, despite numerous fundamental studies on phase-change phenomena^13–16^, the phase transition path has yet to be fully understood, and the many attempts to increase the energy efficiency of phase transition have failed to sufficiently reduce power consumption during actual device operation. Therefore, further studies are being conducted to understand the fundamental switching mechanism and propose new types of phase change materials.

In particular, the phase change process of GeTe with a simple crystalline structure as a typical GST material has been investigated extensively. GeTe, like compounds of any other covalent elements of column V, VI, and VII, is bonded through p electrons with strong directionality forming the perpendicular lobes of p orbitals. Tellurium has a specific characteristic in terms of the number of p-bonding states that combine with another element, such as Ge, with a fewer electrons than itself. Moreover, in this situation, one electron of Te migrates to Ge and an average of five electrons per atom are present in s and p orbitals, satisfying the octet rule and forming three coordinations^17^. In the case of a simple cubic structure, the half-filled p-bands system lowers energy by delocalizing the bonding orbitals via resonant bonds and by opening a gap near the Fermi level through directional bond splitting called Peierls distortion^17^. This coexistence of the Peierls distortion and resonant bonding plays an important role in the phase transition, including photoassisted structure transition and its applications^18–23^. In addition to these structural characteristics, many efforts have been made to improve the understanding of the material itself by applying strain to the material, and further to improve the characteristics of the PRAM^24–27^. In particular, the strained superlattice structure based on layered 2D crystals of GeTe and Sb_2_Te_(x)_ shows enhanced switching properties such as switching speed and energy consumption^25,26^. On the other hand, previous reports have been unable to demonstrate the effects of the experimental application of strain on phase change as such an analysis would require sophisticated thin-film engineering.

In this study, we have investigated the effects of uniaxial stress on directional structure instability and resonant bonding in preferably oriented GeTe (GST prototype) through the controlled bending of a GeTe film. The results show that uniaxial stress can induce a continuous bond interchange between a long bond and a short bond; this can be interpreted as follows: the out-of-plane direction is rotated by an acute angle in the x-axis direction from the z-axis direction. The interface appearing between two different phases may cause a drastic change in optical and electrical properties. This discovery reveals important information on a specific phase-change-like(PC-like) process. First, the data provide some clue about why only AIST shows bond interchange that breaks the long-range directional ordering. For GeTe, which has a relatively smaller difference between short bonds and long bonds compared with AIST, long bond rupture is required to break the long-range order through subsequent lattice relaxation after bond interchange. Second, the most important process in the non-thermal phase transition of phase-change materials is the control of the lattice relaxation process after the initial perturbation rather than the method inducing the perturbation. Without appropriate lattice relaxation process for the resonantly bonded crystal, structures with similar local coordination are preferred over intermediate structures with other local coordination.

## Results and Discussion

**Figure 1:**
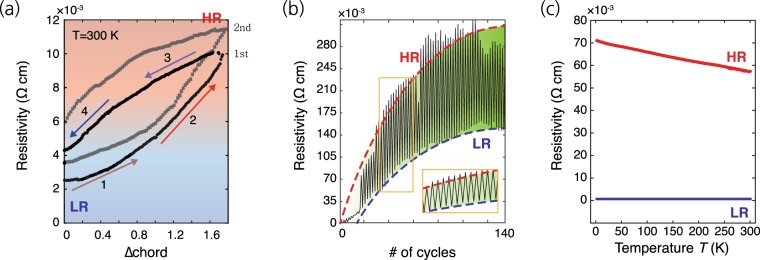
(**a**) Reversible Rs change versus shortened chord length. Rs is irreversibly increased by residual stress in the Polyimide substrate. (**b**) Rs change as a function of bending cycles. The resistivity continously increases under increasing cycles number and show reversible characteristics under bending stress after saturated resistivity region. (**c**) Temperature dependent resistivity from 2 to 300 K. The strained states (The red solid line) show metal-insulator transition behaviour(negative slope).

We investigated changes in electrical properties upon application of reversible bending stresses in well oriented rhombohedral crystalline GeTe (*r*-GeTe). Typically, the stress component that is valid in the grains can be converted to lattice strain with same crystal structure or defects formation^24,28^. Sometimes, phase change process can be observed under a very large stress or large structural instability^29–31^. Figure 1(a) shows the electrical resistivity changes of *r*-GeTe during one-cycle bending. The electrical resistivity of *r*-GeTe increases (decreases) continuously with increasing (decreasing) applied stress. If the basic crystal structure is maintained, a key factor in changing the electrical resistivity in crystalline phase-change materials can be the irreversible defects density, including point defect density and line defect density corresponding to randomly populated vacancy defects rather than a change in the electronic structure^15,16,32^. When performing one cycle of stress application and relaxation, the resistance change can be divided into two sections during the cycle as shown in Fig. 1(a). We define the chord as the modified specimen length along in-plane direction that induces strain on the substrate and delta chord as reduced chord length. The section corresponding to strain application can be further divided into two: (1^st^ and 2^nd^) according to the resistance change rate to the delta chord. The rate increases slowly at the beginning and is faster in the 2^nd^ step. The section corresponding strain relief can also be similarly divided into two steps (3^rd^ and 4^th^) as per the rate of resistance reduction. Although the elastic energy is proportional to the square of the lattice strain, the Δchord does not exactly reflect the change in the lattice strain. Thus, the increment of the strain rate decreases with the Δchord, and the rate of change in the associated physical property also reflects the relationship. Considering this relationship, the resistance change without any saturated behavior cannot be explained by the lattice strain; rather, such a change is much more likely to be caused by the PC-like process occurring at the critical stress. Returning to the zero chord, the resistance may not be fully restored even after the residual stress has disappeared. This may be attributed to the increase in the fraction of transformed phase (phase 2) and/or accompanying disordering. Depending on whether the resistance change is due to a phase change or disorder formation, repeated changes are possible or impossible under the strained and unstrained process: phase change may be a reversible process, while disordering process is not. Further, the resistivity of the strained (high) and unstrained (low) states increases gradually as shown in Fig. 1(b). Finally, after over 100 cycles, the resistivity of two states saturates, resulting in constant resistivity width between two states. This means that the formation ratio of the two phases is constantly maintained through the PC-like process process by the strained and unstrained cycles. The resistance versus Δchord measured in the second cycle is very similar to that in the first cycle. Only the increase in the resistance values of LR and HR after the second cycle was observed, which is consistent with the increased resistance values of the two regions with the number of cycles. However, evidence for the reversible phase transition was clearly observed, which can be confirmed through spectroscopy-based measurements, as shown in Fig. 1(b). The temperature dependence of resistivity can provide valuable information on the PC-like process and disordering, because the scattering behavior of charge carriers depends on the temperature. Figure 1(c) shows temperature-dependent resistivity of strained and unstrained *r*-GeTe films on a polyimide sheet. The unstrained *r*-GeTe film shows a positive temperature coefficient of resistance (TCR): i.e., the resistance decreases linearly with decreasing temperature to 2 K. In contrast, the strained *r*-GeTe film shows a negative TCR. Although the resistivity of the strained *r*-GeTe does not show divergence tendency even at 2 K, a transition from negative TCR to positive TCR is typically occurs near the Anderson localization limit^33,34^: i.e., 0.2 Ω·cm is derived from the Ioffe-Regal condition, as in previous reports^16,32^. Considering the increased number of scattering centers, the scattering mean free path (*λ*_*e*_) is smaller than the electron wavelength (*λ*_*F*_). In this condition, extended Block states can be broken and insulating behavior exists at the localized electronic states condition (*λ*_*e*_*k*_*F*_ < 1), which can be regarded as disorder-induced metal–insulator transition at some points. Previously reported data showed that a great number of disorderings determined by stoichiometric composition can induce clear metal–insulator transition^16,32,35^. Moreover, the defects can be a disordering source for metal–insulator transitions^35^. Even a single-crystalline GeTe nanowire showed a metal–insulator transition induced by extended defects, and this transition is modulated by heat and an intense electric field^35^. Likewise, local disorderings accompanied by phase transition may induce a change in the resistivity characteristics besides the electronic structure difference between two phases. This type of phase transition is also very important, but we consider it to be a different type of PC-like process because we have not applied electrical or thermal energy. Reversible optical transmittance data using Terahertz Time Domain Spectroscopy were also obtained, as shown in Fig. S5. Under uni-axial stress, transmittance value increases steadily and then increases sharply in the last step. Under the decompressing process, transmittance value decreases sharply in the first step and then decreases steadily. Residual increment in transmittance also originates from disordering. It is considered to be the same type of PC-like process as mentioned as observed in this study.

**Figure 2:**
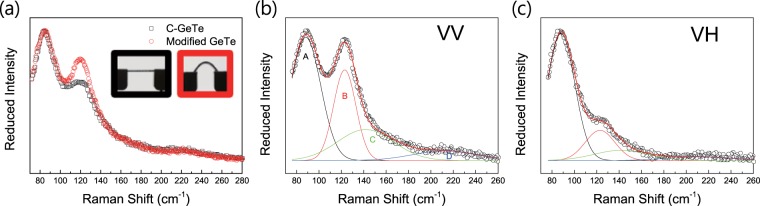
(**a**) Reduced non-polarized Raman spectra of c-GeTe and modified GeTe under uni-axial stress at room Temperature. The inset shows the image of flat(left) and bent(right) 200 nm GeTe film on 1 mm polyimide sheet. The length of substrate in left inset is 2 cm. (**b**,**c**) Reduced polarized Raman spectra of modified GeTe under uni-axial stress at room temperature with different polarization conditions. The excitation and detection polarizations either parallel (VV) or perpendicular (HV).

The local structure in flat and bent GeTe films was analyzed through non-polarized and polarized Raman spectroscopy as shown in Fig. 2. The non-polarized Raman spectra of the c-GeTe film shows two main peaks (Fig. 2(a)). Under uniaxial stress, without significant peak position change and additional Raman active mode, we observe a change in the intensity ratio between the two main peaks corresponding to E_g_ and A_1g_ modes in the rhombohedral structure. Generally, the structure modification by external perturbations such as electromagnetic waves and thermal energy effectively leads to a peak shift or additional peak formation in the Raman spectra^36–39^. This type of modified structure typically becomes a disordered phase without long-range ordering, having an additional Raman active mode for bending and vibrational motion under the specific local structure symmetry. Considering that the Raman modes above 190 cm^−1^ for tetrahedral local structures are hardly changed, the phase deformation under uniaxial stress is not relevant to the conventional phase transformation such as melt-quenched process in GeTe based phase-change materials. According to numerous studies for modified c-GeTe, the second main peak around 120 cm^−1^ representing the A_1g_ mode is caused by the actual mixture with distorted and defective octahedral structures. Since a significant change in the local symmetry and bonding orbital configuration is caused by the difference in the distorted and defective octahedral structures, the enhancement of the second main peak might be originating from drastic structural deformation. To clearly confirm the structural variation, further investigation of the Raman bands in two different configurations were performed using the reduced Raman peak with polarizations for either parallel (VV) or perpendicular (HV) to each other. The spectra of the bent GeTe film under different polarization modes show four Gaussian contributions that are composed of two main bands (A, B) and two additional broad bands (C, D), as shown in Fig. 2(b,c). The curve fitting of the VV and HV states in the bent GeTe film was conducted based on the same fitting frame with the Gaussian distribution. As shown in the reduced non-polarized Raman spectra, the change in the Raman peak under uniaxial stress is predominant due to the increase in the B peak height relative to the A peak height. The contribution of the B peak to the defective mode or symmetric mode can be investigated by the depolarization factor corresponding to the absolute peak area ratio of B peak between VV and HV states^40^. The obtained depolarization factor of B peak is ~0.4, which corresponds to symmetrical vibration^41^ or contains only a very small percentage of symmetry broken one. Thus, the increase in the B peak height under the uniaxial stress is not caused by the structural deformation related to the defective octahedral structure but by the other kind of structural deformation maintaining the local structure. In addition, there is no remarkable change in the Raman modes around 160 cm^−1^ corresponding to one of the vibrations modes originated from the defective octahedral structure as shown in non-polarized Raman spectra (Fig. 2(a)). These results strongly indicate that there is no conventional intermediate phase in laser-irradiated or electrically perturbed specimens^36–39^. Therefore, we suggest that the local orientation change induced by bond interchange can be a cause of the reduction in the Raman scattering cross-section of the E_g_ mode, which is an in-plane vibration mode along the <111> direction in the rhombohedral unit cell. As seen in the AIST system^3–5^, the bond interchange between the short and long bonds can induce the breakage of the long-range ordering.

**Figure 3:**
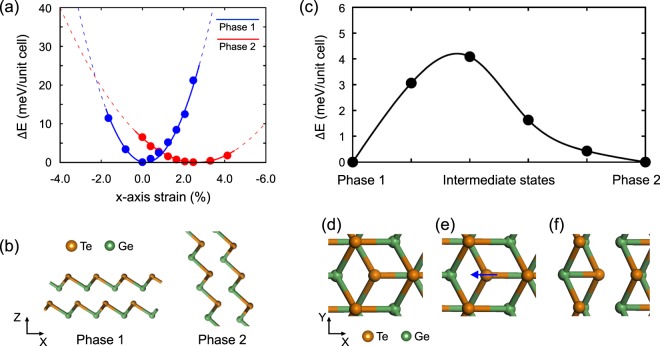
(**a**) Relative energy of Phase 1 (*r*-GeTe; blue line and points) and Phase 2 (modified or rotated GeTe; red line and points) as a function of strain along x-axis while maintaining their phases. Energy values were fitted with a quadratic function. These fitting functions were extended with the dashed lines for their overall trends. (**b**) Equilibrium structures of Phase 1 and Phase 2 corresponding to respective minima in blue and red curves in (**a**). Phase 2, which is another stable structure, can be interpreted as a rotated structure by a certain acute angle. (**c**) Relative energy changes during PC-like process from Phase1 to Phase2. Four intermediate structures are generated from NEB method. Relative energy ΔE shown in (**a,c**) is the total energy per unit cell of each of the corresponding structures in terms of the total energy of the most stable structure. (**d**–**f**) Diagram of PC-like process. (**d,f**) depict Phase1 and Phase2 configurations and four intermediate configurations are compressed into (**e**).

For further investigation, we performed density functional theory (DFT) calculations for the rhombohedral GeTe under uniaxial stress. Crystalline *r*-GeTe can be regarded as a trigonal layered structure consisting of short Ge-Te bonds separated by long Ge-Te bonds. We have designed a structure in which the out-of-plane direction is aligned with the z-axis and the combined direction (**a**_1_ + **a**_2_) of two planar lattice vectors **a**_1_ and **a**_2_ is aligned with the x-axis. To discover possible structural phases of GeTe under an applied external stress and the PC-like process between them, density functional theory (DFT) calculations were performed to scan the whole energy surface as a function of cell parameters. The related cell parameters are shown in Table S6. Our results revealed that there exist two equilibrium structures or two phases, Phase 1 and Phase 2, in the whole energy surface that we calculated. To investigate the structural stability of two phases, we carried out a series of energy calculations as a function of in-plane lattice constants or x-axis strains near their respective equilibria. Figure 3(a) shows two relative energy curves as a function of strain along the *x* direction: one (blue curve) is based on data obtained under the constraint of Phase 1 configuration, and the other (red curve) under the constraint of Phase 2 configuration. Note that the zero strain and energy values were adjusted with respect to those values of the equilibrium Phase 1 structure. Figure 3(b) shows the equilibrium structures of Phase 1 and Phase 2 at their respective energy minima of two curves. It is noted that the Phase 2 is in equilibrium at 2.47% of a tensile strain, but with a compression along the z-axis direction, as compared to Phase 1. However, we found that Phase 2 is just Phase 1 rotated by 71°, maintaining its rhombohedral symmetry with the same volume.

To confirm the specific correlation between structural deformation and PC-like process, we evaluated the energy barriers of the phase transition between two structures on blue (Phase 1) and red (Phase 2) curves using nudged elastic band (NEB) method. For this evaluation, we generated four intermediate states between them and calculated their corresponding energies, as shown in Fig. 3(c). It was found that Phase 1 undergoes the phase transition under uniaxial tensile stress by overcoming an energy barrier of 4.20 meV/unit cell. This energy barrier is much larger than the energy value (~2.49 meV/unit cell) at which two phases cross in Fig. 3(a). Although the two phases cross at the same point in the figure, their respective corresponding structures are quite different since they are under the constraint of keeping their phases.

From the view point of local coordination, the only difference between Phase 1 and Phase 2 is the bond-orientation of three short bonds and three long bonds. As shown in Fig. 3(d–f), when the deformation is applied to Phase 1 along the x direction, the central Te atom is displaced: i.e., eventually one of short Ge-Te bonds is converted to a long bond and the central Te atom forms a short bond with a Ge atoms located at the upper layer, which acted as a long bond before the deformation, as clearly seen in Fig. 3(d–f). It can be interpreted that the out-of-plane direction has been rotated by an acute angle from the z-axis towards the x-axis direction. Simply phase transitions may be possible through a coupling exchange between one short bond and one long bond. Both phases are rhombohedral with the space group of R3m.

So far, our analysis on the PC-like process properties according to the structural transformation is based on the unit structure in which only two atoms, one Ge and one Te atoms are considered. We used infinite repetition of the unit cell in our DFT calculations meaning that intermediate states considered between Phase 1 and Phase 2 are also infinitely repeated, but the energy values considered above were all given in per-unit cell. Thus, it requires a lot of energy to induce PC-like process in a large area. In addition, the well-oriented rhombohedral crystalline structure considered in our calculations is a model representing a crystalline domain in a polycrystalline sample. Under our experimental conditions, we inferred that initially only a few domains may experience bond switching while the others may not, but more domains would experience bond switching under repeated bending process. Moreover, our calculations were performed in small unit cells containing no imperfections. To check the effect of defects, we actually tried extended model calculations to better fit to experimental conditions. To explore the mechanism of the local phase transition, we prepared a model structure of the 3 × 3 × 3 supercell consisting of 27 Phase 1 configurations to study the phase transition to phase 2 under the uniaxial stress. We carried out NEB calculation by converting Phase 1 to Phase 2 configurations one unit by one unit. The energy barrier of the phase transition from Phase 1 to Phase 2 was evaluated to be more than 0.6 eV, which is much larger than the value evaluated in Fig. 3(c). Such a large energy barrier was assigned to unbalanced ratio of short Ge-Te bonds to long Ge-Te bonds, which should be balanced to be 3:3 in the crystalline *r*-GeTe. During the local PC-like process, such balanced ratio becomes unbalanced to be either 2:4 or 4:2. It was found that the whole 3×3×3 supercell becomes stabilized more towards Phase 2 when Phase 1 configurations in 18 or more units are converted to Phase 2 resulting in reduction of the unbalanced ratios, thus both Phase 1 and Phase 2 may be distributed in the form of grains in the strained GeTe structure of more than 1.0%.

**Figure 4:**
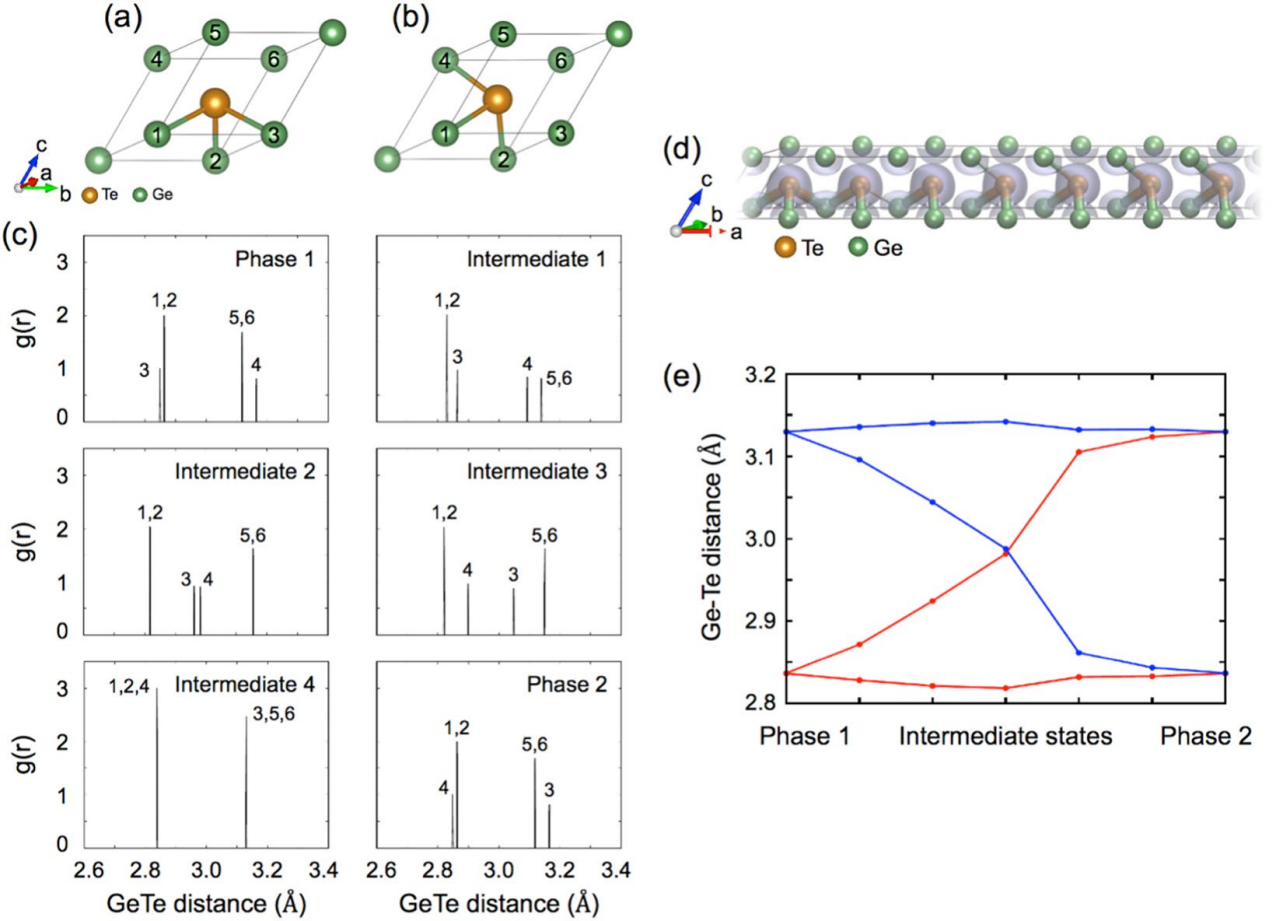
Local unit structure of Phase 1 (**a**) and Phase 2 (**b**). (**c**) Evolution of the radial distribution functions (RDFs) during the PC-like process between Phase 1 and Phase 2 at 2.0% of an uniaxial strain along the x-axis. The numbers marked at peaks in RDFs correspond to the bonds formed between the central Te atom and the same numbered Ge atoms in (**a,b**). (**d**) Interfacial model structure between Phase 1 and Phase 2 overlapped with the charge density difference showing bond switching at the interface. (**e**) Change in the bond distances of six nearest neighboring Ge-Te bonds at the interface between Phase 1 and Phase 2.

We carefully interpreted the phase change mechanism based on intermediate structures revealed through our NEB calculations as well as comparison of stress induced PC-like process with those of conventional GST and AIST system. Under uni-axial stress, the PC-like process through mutual interchange between short and long bonds can be thought as a three-step process as shown in Fig. 4. Initially, Phase 1 consists of three short bonds, which are formed by the central Te atom connected with three intraplanar neighboring Ge atoms #1, #2, and #3, and three long bonds formed by the central Te atom with three interplanar neighboring Ge atoms #4, #5, and #6, as shown in Fig. 4(a). In the first step, when tensile stress is applied in the x direction, which also plays a part in compressive stress along the z direction, two of its three long bonds are elongated further without shortening the short bonds, as shown in Fig. 4(c), because majority of the stress is used to deform long bonds due to high bond-energy hierarchy between short and long bonds. This deformation is also valid for uni-axial stress in either direction because at least one long bond be under influence by this type of tensile stress. This structural change maximizes the difference between the short bond length and the long bond length, which is indicated by Peierls distortion. Because of the limited electronic energy gain that can be achieved with Peierls distortion, the rapidly increasing elastic energy makes the structure unstable. A.V. Kolobov study showed that without additional external perturbation, this severely distorted structure could be transformed to disordered phases^12^. On the other hands, in a uniaxial stress environment, another type of transition can occur with the help of residual stress. Especially, this characteristic is caused by delocalized bonding orbital of GeTe known as resonance bond. The second step involving the bond-interchange process represented by Intermediate 2 through Intermediate 4 structures proceeds as a preprocessing step to restore the resonance bond. Continuous tensile stress along the x direction and thus compressive stress along the z direction generates a bond between the central Te atom and Ge #4 atom depicted in Fig. 4(b). This progress simultaneously induces the weakening of the bond between the central Te atom and Ge #3 atom, and eventually one long bond and one short bond exchange as shown in RDF analysis of Fig. 4(c). This tendency implies a possibility of recovering resonant bond without tetragonal or defective final state. The third step is caused by distortion mitigation, resulting in the increased instability. As the bond interchange occurs, the uniaxial stress is no longer effective for deforming the structure due to the revolved crystal axis. At this time, the process of recovering the local structure of the initial state is performed.

Moreover, we exploited the interfacial features between two phases. We generated a model consisting of Phase 1 at the left end, Phase 2 at the right end, and five unit structures mimicking the interface region between the two phases, as shown in Fig. 4(d). We performed the geometrical relaxation on the Te atoms only in the interface regions while keeping the Te atoms at both ends fixed. As a result, it was observed that the long bond and the short bond becomes apparently interchanged, as shown in Fig. 4(d). Additionally, the evolution of the charge density difference during the PC-like process indicates how the resonance bonding character disappears and is being restored as shown in Fig. 4(d). Figure 4(e) shows the trend of the length change of three short bonds and three long bonds during the PC-like process between Phase 1 and Phase 2. It clearly displays the bond interchange between one short and one long bond, whereas the other four bonds retain their bonding character. It is noted that such interface structures may not exist independently by themselves due to its structural instability, but it can inevitably exist at the interface between the two phases. In addition, because resistance changes due to structural defects in phase change materials typically change toward increasing resistance, although we did not collect any data about the negative chord, the increasing resistance can be inferred by considering the phase transition mechanism.

**Figure 5:**
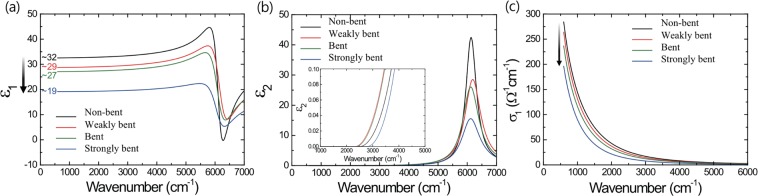
Infrared Spectroscopic Ellipsometry result for crystalline GeTe depending on the variation of uni-axial stress with four degrees as non-bent(black), weakly-bent(red), bent(green), and strongly-bent(blue) GST. (**a**) Real part (ε_1_) and (**b**) imaginary part (ε_2_) permittivity spectra in Tauc-Lorentz term show the comparison of zero frequency optical dielectric constant ε_1_(0) levels and interband transition with approximation of parabolic bands under degrees of stress, respectively. (**c**) Real part conductivity spectra related to free carrier response estimated by Drude model show large degradation associated with disorder-induced localization under the uni-axial stress in GT.

Depending on the size of the PC-like process from Phase 1 to 2 via bond interchange, long-range ordering can be disrupted by the p-orbital misalignment near the interface. Micro-ellipsometry was employed to investigate the change in dielectric properties caused by p-orbital change about the far-IR range from 0.075 to 0.87 eV. In this range, the dielectric function is composed of contributions from valence electrons and free electrons because of irrespective contributions from phonons with 0.04 eV highest value. The high-frequency optical dielectric constant *ε*_1_(0) is defined as the value of the real part of the dielectric function at the low energetic limit excluding the Drude contribution in this region, as shown in Fig. 5(a). *ε*_1_(0) is a macroscopic optical characteristic that includes only polarizability characteristics of valence electrons, it can reveal bond properties, e.g., delocalization characteristics that lead to larger electronic displacements upon external electromagnetic excitement. Thus, the circumstantial data for processing procedure can be explained in the method. Upon variation of the uniaxial stress strength, *ε*_1_ and *ε*_2_ of rhombohedral GeTe (*r*-GeTe) were compared with those of bond-interchanged GeTe (BI-GeTe), shown in Fig. 5(a,b), respectively. *ε*_2_ of BI-GeTe is smaller than that of *r*-GeTe, indicating that *r*-GeTe can transition into BI-GeTe as per Kramers-Kronig analysis. *ε*_1_(0) of *r*-GeTe, about 32, gradually decreases to 19 upon uniaxial stress application. The particularly drastic change in *ε*_1_(0) from 27 to 19 at the final step is caused by the application of stress over a critical point during the bond interchange, as shown by the calculation data. Although this *ε*_1_(0) value is higher than the amorphous one, it is much lower than that corresponding to other modified crystalline structures. As per the Penn gap rule by Shimakawa *et al*.^42^, the increase in the high-frequency dielectric constant upon crystallization in phase-change materials may not be relevant to the resonance property but can be relevant to the decrease in the peak energy E_0_ in *ε*_2_. Because there is little change in E_0_ in this case, the drastic change in the high-frequency dielectric constant should be attributed to a matrix element governed by the special bonding arrangement producing the resonance effect. The low-energy area of the dielectric function containing *ε*_1_(0) is governed by the electronic polarizabililty of the valence electrons: i.e., the dielectric function is given by the product of a matrix element and the integral over the joint DOS. In *r*-GeTe, the dipole matrix elements M = <i/x/f> are very large due to well-aligned resonant bonds, where i and f are the initial and final states. For normal bonds between two atoms, M has the value of about one bond length, while for resonant bonding, M has the value of two typical bond lengths due to the aligned chains. This large dipole matrix element induces a large *ε*_1_(0), as shown in Fig. 5(a). Sufficient calculation results show that this large *ε*_1_(0) in GeTe can be rapidly decreased by losing medium-range ordering, without a significant change in the DOS. Simply, misaligned p orbitals can induce the breakage of medium-range order, while the coordination number is maintained except at the interface between *r*-GeTe and BI-GeTe. Considering the similarity of DOS between *r*-GeTe and BI-GeTe, the decrease in optical dielectric constant down to 40% is not caused by the change in DOS. Instead, it is more acceptable that the BI structure generated in the R3m structure causes misalignment, which breaks p-orbital resonance bonds and gives rise to a decrease in the dipole matrix elements. Considering all the experimental results together, grain-to-grain interface and disordering might affect the decrease in resistivity. If the stress in the inter-grain boundary is slightly relaxed, there would be little change in the Raman spectra. On the contrary, if a new chemical bond is formed on the inter-grain boundary with larger stress, a new peak originating from specific local structure should be seen in Raman spectra. In other words, peaks appearing at over 150 cm^−1^ which appears in intermediate structure of conventional phase change materials, such as pyramidal structures and defective octahedra structures, should be seen^36–39^. Additionally, the new specific changes induced around the intrinsic defect as well as the formation of the new local structure should be also visible to other well-known peaks. However, peaks due to the new local structure were not seen and only the difference in peak intensity between a_1g_ and e_g_ mode was only seen. Therefore, although the changes induced by grain boundary and defect can also affect the bending process, the effect is not considerable. Moreover, there is no reason to rapidly decrease the dielectric constant even if there is a new bond at the interface where the resonant bonds are already broken. Therefore, it can be concluded that the optical changes strongly supports the structural change.

## Conclusion

We have demonstrated that both directional structure distortion and resonance bonding can induce non-thermal PC-like process via bond switching between long and short bonds and the subsequent collapse of bonding orbital alignment. The concomitant change in electrical, optical, and structure properties were investigated for rhombohedra GeTe under bending stress. DFT calculation results suggest that the PC-like process through bond switching progresses in three steps: distortion triggering owing to bonding energy hierarchy, bond switching as a preprocessing step to restore the delocalized bond, and distortion relaxation. Until now, the PC-like process through bond interchange has not been observed in the GST group (GeTe-Sb_2_Te_3_ pseudo-binary alloys). Moreover, bond interchange observed in the AIST system (Ag, In-doped SbTe alloys) could be through thermal perturbation. From this perspective, our investigation gives some important information on specific PC-like process. The data indicate why only AIST shows bond interchange resulting in breakage of long-range directional ordering. For GeTe with smaller difference between the short and long bonds than AIST, long bond rupture is required to break the long-range order through subsequent lattice relaxation after bond interchange. In addition, for non-thermal PC-like process in phase-change materials, the data show that the most important process is the control of the lattice relaxation process after initial perturbation and not the method inducing the perturbation. The bond switching has not been considered yet as a factor in thermodynamic perturbation. However, from the PC-like process by bond interchange, we conclude that it can be an important variable for subsequent lattice relaxation in phase-change materials. The suggested non-thermal mechanism would be applicable to optical phase change device because long-range order breakage through the non-thermal process changes dielectric property

## Method

### Material synthesis and stress application

A GeTe film (200-nm-thick) was deposited on a polyimide (PI) substrate (1 mm) at room temperature via ion beam sputtering deposition using a single GeTe target. For acquiring the crystalline phase, the annealing process was conducted in a N_2_ ambient using a rapid thermal process (RTP), where the isothermal duration was 1 h at 250 °C, which is greater than the crystallization temperature. The atomic concentrations and contaminant elements were analyzed by x-ray photoemission spectroscopy (XPS) and the rhombohedral crystalline phase were confirmed by x-ray diffraction (XRD) analysis. Uniaxial stress was applied using the home-built bending system. By bending the PI substrate containing a deposited GeTe film, uniaxial stress (tensile stress along in-plane direction, compressive stress along out-of-plane direction) was generated on the GeTe film on the stretched PI substrate. To apply a large stress on the GeTe film, we used a thicker substrate to stretch the outside arc longer. Because the flexible polyimide substrate does not contain any crystalline ordering and the GeTe film has low thermal desorption temperature, it was difficult to synthesize a single crystalline film. Nevertheless, we successfully obtained poly crystalline structure with the preferred orientation in the specific growth direction (202), using ion beam sputtering, as shown in Fig. S4 because the ion beam effectively transfers energy to atoms impinging on substrate. The intended experiment could be performed adequately using this thin film. For a very special case like GeTe which has directional structural instability such as 3D-like Peierls distortion^18^, well-preferred oriented polycrystalline structure might be effectively affected by uni-axial stress^43^. Moreover, because of the bonding characteristics caused by the Peierls distortion, the bonding direction can be easily aligned to the stress direction. Although the expected in-plane tensile strain for 1.2 mm delta chord is about 3%, due to young’s modulus difference between film and substrate, adhesion property, and intergrain relaxation, we can infer the value of in-plane tensile strain to be about 1% which is consistent with DFT results.

### Density functional theory calculation

To investigate the structural stabilities and the electronic properties of GeTe, we used density functional theory (DFT)^44^. Electronic wavefunctions were expanded into plane wave basis with a cutoff energy of 400 eV, and the ion–electron interactions were described by the projector augmented-wave pseudopotentials^45^ implemented in the Vienna ab initio simulation package (VASP)^46,47^. The exchange-correlation functional was treated within the generalized gradient approximation (GGA) method in the form of the Perdew-Burke-Ernzerhof (PBE)^48^. Charge density was self-consistently determined with a precision of lower than 10^−5^ eV/cell in total energy. Atomic relaxation was continued until the Helmann-Feynman forces acting on the atoms were lower than 0.01 eV/Å. The corresponding Brillouin zone was sampled using a Γ-centered 20 × 20 × 20 *k*-grid mesh for the primitive unit cell. To better describe the interaction between GeTe layers, we included van der Waals interaction using Grimme’s method (DFT-D2)^49^. To analyze PC-like process between two phases, we applied the variable cell nudged elastic band (NEB) method^50,51^.

### Ellipsometry

In order to extract the complex permittivity function of strained GeTe, Fourier transform infrared spectroscopic ellipsometer (FTIR-SE) that has been upgraded from Sopra GES5 with FTIR setup has been used with range from 0.075 to 0.87 eV at the incidence of 75°. The FTIR light source that has been used in this research is slicon carbide nano rod. As detector, an InSb photovoltaic detector was used for FIR to NIR region with the operation temperature of −196 °C. A micro spot lens was used to minimize errors which were caused by the curved surface in strained GeTe film.

As shown in Fig. S1, the values of α and β related to tanψ and cosΔ of GeTe with strain in the range from 0.075 to 0.87 eV were used to extract a complex permittivity function $\epsilon ({\rm{\omega }})$ which is composed of the main three terms as shown following equation.

$$\epsilon ({\rm{\omega }})={\epsilon }_{\infty }+{\epsilon }_{{\rm{Drude}}}({\rm{\omega }})+{\epsilon }_{\mathrm{Tauc} \mbox{-} \mathrm{Lorentz}}({\rm{\omega }})$$

The 1^st^ term, ${\epsilon }_{\infty }$, is the value of ${\epsilon }_{1}$ at higher energy region near infinite frequency which related with the polarizability of core electron. The 2^nd^ term, ${\epsilon }_{{\rm{Drude}}}({\rm{\omega }})$, is strongly associated with the free carrier response in FIR region which is the well-known Drude model in the case of a crystalline solid specimens.

$${\epsilon }_{{\rm{Drude}}}({\rm{\omega }})=\frac{{{\rm{\omega }}}_{{\rm{p}}}^{2}}{{\rm{\omega }}({\rm{\omega }}+i{\rm{\gamma }})}$$

where ω_p_ and γ are the plasma frequency and electron scattering rate. The Drude parameters of GeTe thin film were well estimated by well fitted the combined model over all measurement region (Fig. S2). The 3^rd^ term, ${\epsilon }_{\mathrm{Tauc} \mbox{-} \mathrm{Lorentz}}({\rm{\omega }})$, is the Tauc-Lorentz oscillator to demonstrate the transition edge of the optical interband transition^52,53^.

$$\begin{array}{llll}{\epsilon }_{2}({\rm{\omega }}) & = & \frac{1}{{\rm{\omega }}}\frac{{{\rm{A}}{\rm{\omega }}}_{0}{\rm{C}}({\rm{\omega }}-{{\rm{\omega }}}_{{\rm{gap}}})}{{({{\rm{\omega }}}^{2}-{{\rm{\omega }}}_{0}^{2})}^{2}+{{\rm{C}}}^{2}{{\rm{\omega }}}^{2}} & ({\rm{\omega }} > {{\rm{\omega }}}_{{\rm{gap}}})\\  & = & 0 & ({\rm{\omega }}\le {{\rm{\omega }}}_{{\rm{gap}}})\end{array}$$

The ${\epsilon }_{1}$ of the Tauc-Lorentz model is given by the Kramers-Kronig relation associated between real and imaginary permittivity as shown following equation.

$$\begin{array}{rcl}{\epsilon }_{1}({\rm{\omega }}) & = & \frac{{\rm{AC}}}{{{\rm{\pi }}{\rm{\xi }}}^{4}}\frac{{{\rm{a}}}_{\mathrm{ln}}}{2{{\rm{\alpha }}{\rm{\omega }}}_{0}}\,\mathrm{ln}(\frac{{{\rm{\omega }}}_{0}^{2}+{{\rm{\omega }}}_{{\rm{gap}}}^{2}+{{\rm{\alpha }}{\rm{\omega }}}_{{\rm{gap}}}}{{{\rm{\omega }}}_{0}^{2}+{{\rm{\omega }}}_{{\rm{gap}}}^{2}-{{\rm{\alpha }}{\rm{\omega }}}_{{\rm{gap}}}})\\  &  & -\,\frac{{\rm{A}}}{{{\rm{\pi }}{\rm{\xi }}}^{4}}\frac{{{\rm{a}}}_{\tan }}{{{\rm{\omega }}}_{0}}[{\rm{\pi }}-{\tan }^{-1}(\frac{2{{\rm{\omega }}}_{{\rm{gap}}}+{\rm{\alpha }}}{{\rm{C}}})+{\tan }^{-1}(\frac{-2{{\rm{\omega }}}_{{\rm{gap}}}+{\rm{\alpha }}}{{\rm{C}}})]\\  &  & +\,\frac{2{{\rm{A}}{\rm{\omega }}}_{0}}{{{\rm{\pi }}{\rm{\xi }}}^{4}{\rm{\alpha }}}{{\rm{\omega }}}_{{\rm{gap}}}({{\rm{\omega }}}^{2}-{{\rm{\gamma }}}^{2})[{\rm{\pi }}+2{\tan }^{-1}(2\frac{{{\rm{\gamma }}}^{2}-{{\rm{\omega }}}_{{\rm{gap}}}^{2}}{{\rm{\alpha }}{\rm{C}}})]\\  &  & -\,\frac{{{\rm{A}}{\rm{\omega }}}_{0}{\rm{C}}}{{{\rm{\pi }}{\rm{\xi }}}^{4}}\frac{{{\rm{\omega }}}^{2}+{{\rm{\omega }}}_{{\rm{gap}}}^{2}}{{\rm{\omega }}}\,\mathrm{ln}(\frac{|{\rm{\omega }}-{{\rm{\omega }}}_{{\rm{gap}}}|}{{\rm{\omega }}+{{\rm{\omega }}}_{{\rm{gap}}}})\\  &  & +\,\frac{2{{\rm{A}}{\rm{\omega }}}_{0}{\rm{C}}}{{{\rm{\pi }}{\rm{\xi }}}^{4}}{{\rm{\omega }}}_{{\rm{gap}}}\,\mathrm{ln}[\frac{|{\rm{\omega }}-{{\rm{\omega }}}_{{\rm{gap}}}|({\rm{\omega }}+{{\rm{\omega }}}_{{\rm{gap}}})}{\sqrt{{({{\rm{\omega }}}_{0}^{2}+{{\rm{\omega }}}_{{\rm{gap}}}^{2})}^{2}+{{\rm{\omega }}}_{{\rm{gap}}}^{2}{{\rm{C}}}^{2}}}]\end{array}$$

where

$$\begin{array}{rcl}{{\rm{a}}}_{\mathrm{ln}} & = & {({\rm{\omega }}}_{{\rm{gap}}}^{2}-{{\rm{\omega }}}_{0}^{2}{){\rm{\omega }}}^{2}+{{\rm{\omega }}}_{{\rm{gap}}}^{2}{{\rm{C}}}^{2}-{{\rm{\omega }}}_{0}^{2}({{\rm{\omega }}}_{0}^{2}+3{{\rm{\omega }}}_{{\rm{gap}}}^{2})\\ {{\rm{a}}}_{\tan } & = & ({{\rm{\omega }}}^{2}-{{\rm{\omega }}}_{0}^{2})({{\rm{\omega }}}_{0}^{2}+{{\rm{\omega }}}_{{\rm{gap}}}^{2})+{{\rm{\omega }}}_{{\rm{gap}}}^{2}{{\rm{C}}}^{2}\\ {{\rm{\xi }}}^{4} & = & {({{\rm{\omega }}}^{2}-{{\rm{\gamma }}}^{2})}^{2}+{{\rm{\alpha }}}^{2}{{\rm{C}}}^{2}/4\\ {\rm{\alpha }} & = & \sqrt{4{{\rm{\omega }}}_{0}^{2}-{{\rm{C}}}^{2}}\\ {\rm{\gamma }} & = & \sqrt{{{\rm{\omega }}}_{0}^{2}-{{\rm{C}}}^{2}/2}\end{array}$$

In the case of Tauc-Lorentz model, a total of four parameters (A, C, ω_0_, ω_gap_) play the role to determine the complex permittivity function above complicated equation $\epsilon ({\rm{\omega }})$. Here, it can be seen from Fig. 5 that ${\epsilon }_{2}=0$ at ω ≤ ω_gap_ and the peak position of ${\epsilon }_{2}$ is ω_0_. The A and C in this model mean the amplitude and half width of the ${\epsilon }_{2}$ peak, respectively. The values of the analytical parameters in this calculation as ${\epsilon }_{\infty }$, ω_p_, γ, A, C, ω_0_, ω_gap_ can be extracted by least square fitting method in home grown software using MATLAB. The extracted parameters in the Drude and the Tauc-Lorentz model were tabulated in the Tables 1 and 2. We have extracted the complex optical constants conductivity using this combined model with complex number.

**Table 1:** The parameters of Tauc-Lorentz model.

	A (eV)	C (eV)	ω_0_ (eV)	${{\boldsymbol{\epsilon }}}_{{\boldsymbol{\infty }}}$	ω_gap_ (eV)
Non-bent	6.60	0.05	0.76	61.89	0.31
Weakly bent	5.93	0.08	0.77	57.25	0.29
bent	5.65	0.08	0.76	49.52	0.29
Strong bent	4.88	0.09	0.76	36.33	0.35

**Table 2:** The paramerters of Drude model.

	σ_DC_(Ω^−1^*cm*^−1^)	ω_p_ (eV)	γ (eV)
Non-bent	3110	2.298	0.229
Weakly bent	2920	2.151	0.213
bent	2650	2.001	0.203
Strong bent	2320	1.697	0.167

### Terahertz spectroscopy

The THz- probe pulses were generated via optical rectification of the fun- damental pulse in a 10 × 10 mm <110> ZnTe crystal with a thickness of 1 mm. The transmitted THz radiation was detected by electro-optic (EO) sampling in another <110> ZnTe nonlinear crystal with thickness of 3 mm. The signal was collected with a lock-in amplifier (SR830, Stanford Research System) that was phase-locked to an optical chopper, which modulated either the THz generation line for TDS. All TDS experiments that were carried out in this study were performed at room temperature and the entire THz beam path was enclosed and purged with dry air. For the extraction of frequency-dependent transmission character- istics, THz-TDS requires two transmitting signals, Esample(t) and Eref(t), with and without the sample, respectively. Fast Fourier transform (FFT) was applied to calculate the trans- mission amplitude and phase in the frequency domain. The amplitude A(ω) of the ratio of the two spectra, Esample(ω)/Eref (ω), was calculated and analyzed to obtain the frequency- dependent transmission amplitude.

## Supplementary information

Phase-change like process through bond switching in distorted and resonantly-bonded crystals
