# Supplementary material for: Phase-change like process through bond switching in distorted and resonantly bonded crystal
Source: Sci Rep. 2019 Sep 6;9:12816. doi: 10.1038/s41598-019-49270-2 (PMC6731313; doi:10.1038/s41598-019-49270-2)
Supplement: Supplementary file 1 — Phase-change like process through bond switching in distorted and resonantly-bonded crystals [file 41598_2019_49270_MOESM1_ESM.pdf]

# Phase-change like process through bond switching in distorted and resonantly-bonded crystals

Won Jun Yang,[1] Hanjin Park,[2] Da Sol Kim,[1] Taewoo Ha,[1†] Seung Jong Park,[1] Min Ahn,[1] Jae Hoon Kim,[1] Young-Kyun Kwon,[2,*] and Mann-Ho Cho[1,*]

[1] *Department of Physics and Applied Physics, Yonsei University, Seoul 03722, Republic of Korea.*

[2] *Department of Physics and Research Institute for Basic Sciences, Kyung Hee University, Seoul 02447, Republic of Korea.*

Fig.S1 Infrared Spectroscopic Ellipsometry

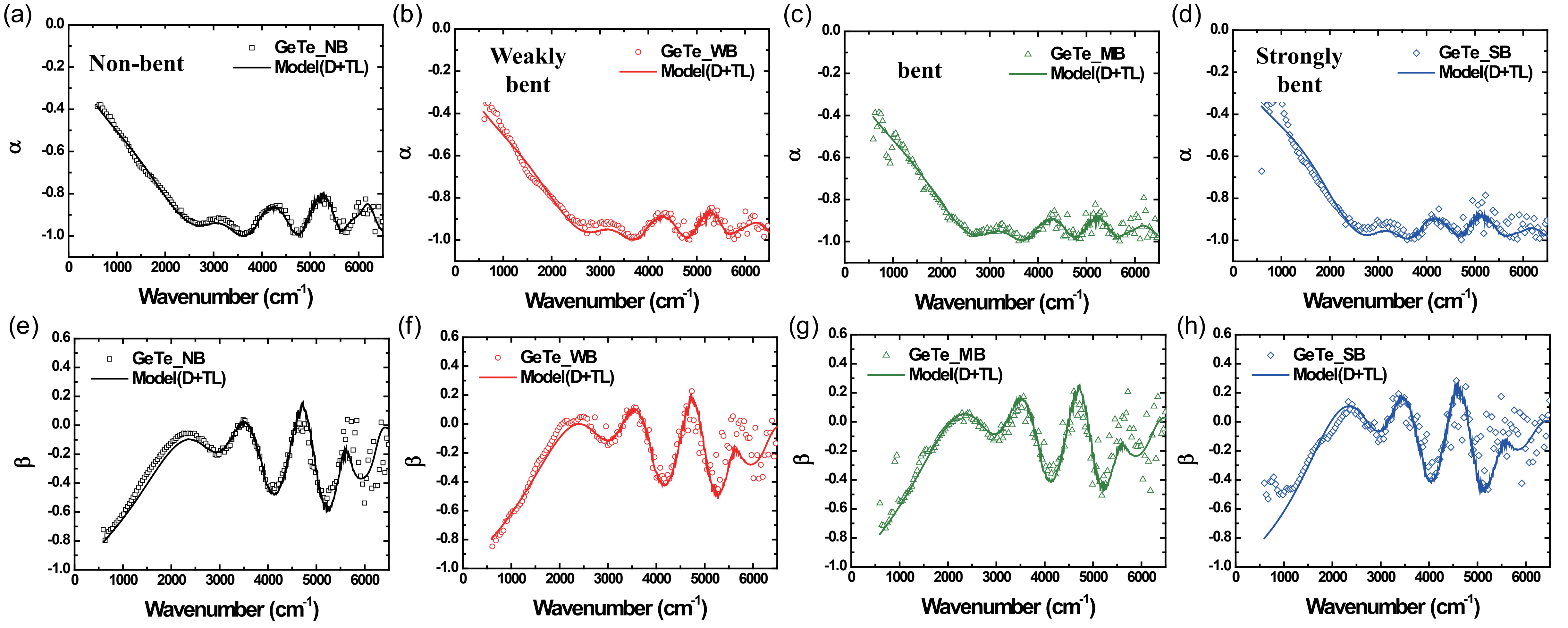


Figure S1 Drude-Tauc-Lorentz model fitting, which the model composed of linear summation with Drude and Tauc-Lorentz model, on $\alpha$ and $\beta$ observed by the infrared spectroscopic ellipsometry under the four uni-axial stressed GeTe as (a)&(e) Non-bent (black), (b)&(f) weakly-bent (red), (c)&(g) bent (green), and (d)&(h) strongly bent (blue). The experimental data and model are expressed by symbol and solid line, respectively.

Fig.S2 Permittivity with Drude-Tauc-Lorentz Model

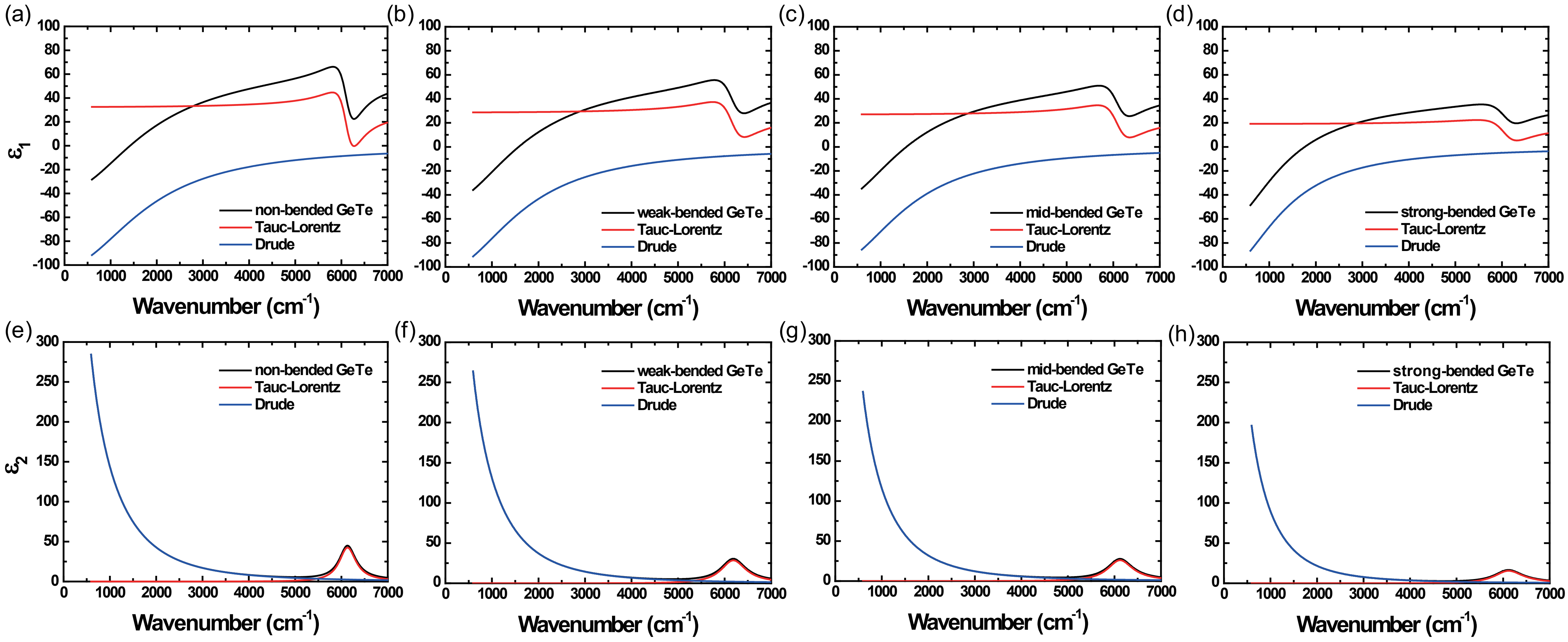


Figure S2 The complex dielectric constant of Drude-Tauc-Lorentz model fitting results. Real part ($\varepsilon_1$) and imaginary part ($\varepsilon_2$) dielectric constant of (a)&(e) non-bent, (b)&(f) weakly bent, (c)&(g) bent, and (d)&(h) strongly bent. In the each graph, Drude model, Tauc-Lorentz model, and total fitting are separately given by solid line with colors as blue, red, and black, respectively.

Fig.S3 Energy barrier during the phase transtion

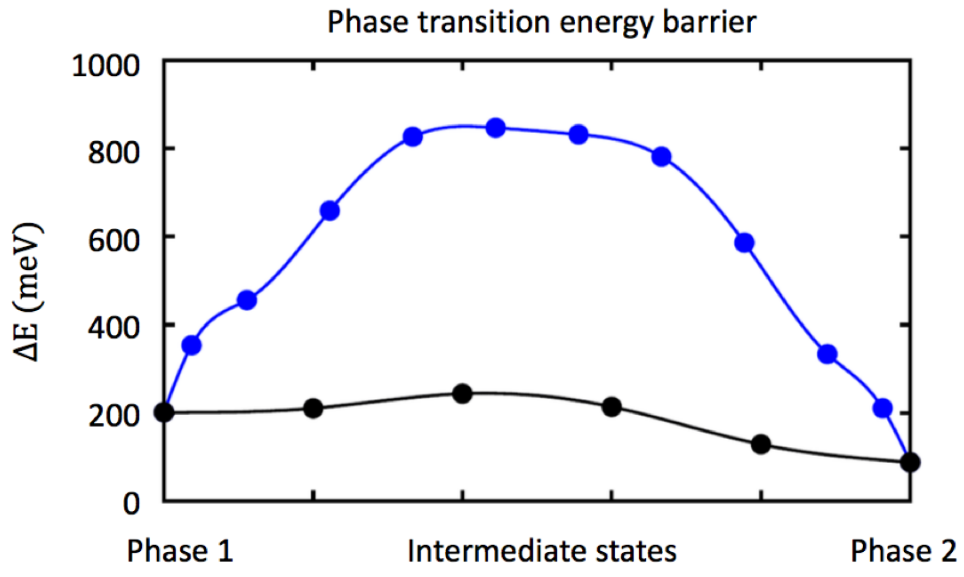


Super cell (3x3x3)

Figure S3. Energy change during the phase transition between Phase 1 and Phase 2 constructed in 3×3×3 supercell. The blue curve shows the transition energy barrier of the unit-by-unit phase transition from Phase 1 to Phase 2, while the black one displays energy change during the phase transition of the whole 27 units simultaneously.

Fig.S4 X-ray diffraction Spectra

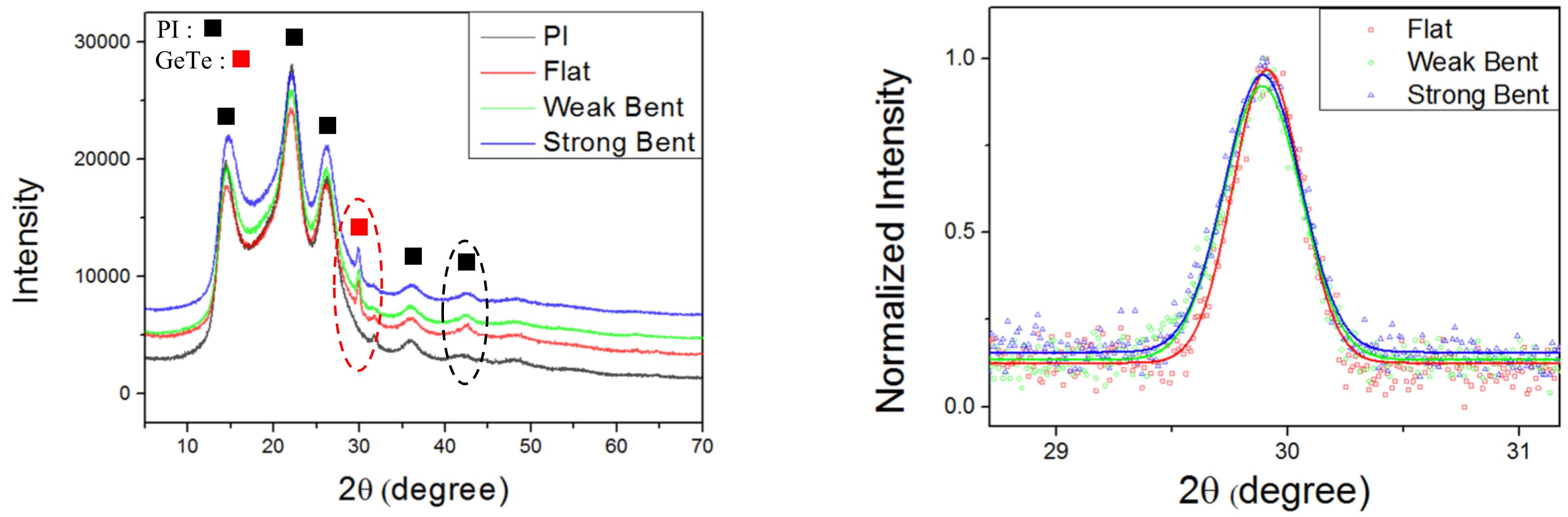


Figure S4. XRD experiments performed in theta-2 theta geometry. Black line on the left side corresponds to polyimide substrate information; the other lines represent GeTe film on substrate upon uniaxial stress. A peak due to the thin film appears at approximately 30°, which means that the thin film is oriented toward the (202) direction. The peak in the vicinity of 42.5° is relatively very small, compared with the main (202) peak. Thus, we consider the (202) crystalline plane in the phase-change-like process, and not the (024), (220) crystalline plane. At the (202) peak, the change in peak positions under uniaxial stress is negligible, while that in FWHM was clearly observed. From the Scherrer equation, the grain size decreased by 12% related to increased interface by layer rotation.

Fig.S5 The real part of optical transmittance data of GeTe film from Terahertz Spectroscopy

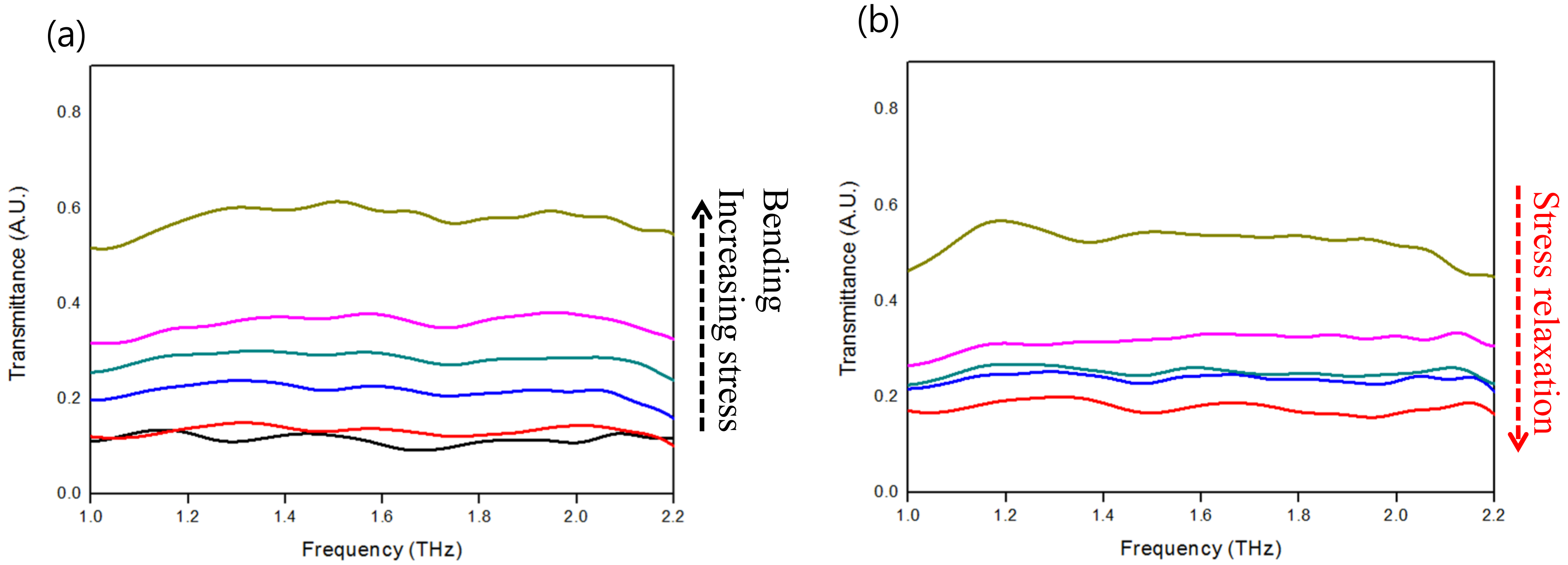


Figure S5 The real part of optical transmittance calculated from Terahertz Time Domain Spectroscopy Black line in (a) corresponds to spectra of unstressed film. Under uni-axial stress, Transmittance value of GeTe film increases under uni-axial stress as shown in (a). It is closely related to decrease of resistivity as shown in Fig (1). Transmittance value of GeTe film also decreased under stress relaxation as shown in (b). Reversible change is originated from reversible structure property which is explained as bond switching driven layer rotation.

Table.S6 Cell parameter

| Phase 1 | x | y | z | Intermediate 3 | x | y | z |
|---|---|---|---|---|---|---|---|
| a | 3.6151 | -2.1068 | 0.0000 | a | 3.7801 | -2.1047 | 0.0000 |
| b | 3.6151 | 2.1068 | 0.0000 | b | 3.7732 | 2.1198 | 0.0000 |
| c | 2.4100 | 0.0000 | 3.4623 | c | 2.6362 | 0.0055 | 3.5176 |
| Intermediate 1 | x | y | z | Intermediate 4 | x | y | z |
| a | 3.7178 | -2.1054 | 0.0000 | a | 3.8072 | -2.1039 | 0.0000 |
| b | 3.7139 | 2.1088 | 0.0000 | b | 3.7921 | 2.1337 | 0.0000 |
| c | 2.4397 | 0.0045 | 3.6204 | c | 2.5888 | 0.0098 | 3.4638 |
| Intermediate 2 | x | y | z | Phase 2 | x | y | z |
| a | 3.7178 | -2.1054 | 0.0000 | a | 3.6874 | -2.1032 | 0.0000 |
| b | 3.7139 | 2.1088 | 0.0000 | b | 3.6874 | 2.1032 | 0.0000 |
| c | 2.5777 | 0.0045 | 3.5733 | c | 2.4582 | 0.0000 | 3.4429 |

Table S6. Cell parameters of Phase 1, Phase 2, and 4 intermediate structure as a result of NEB method.

Fig.S7 Bending module image.

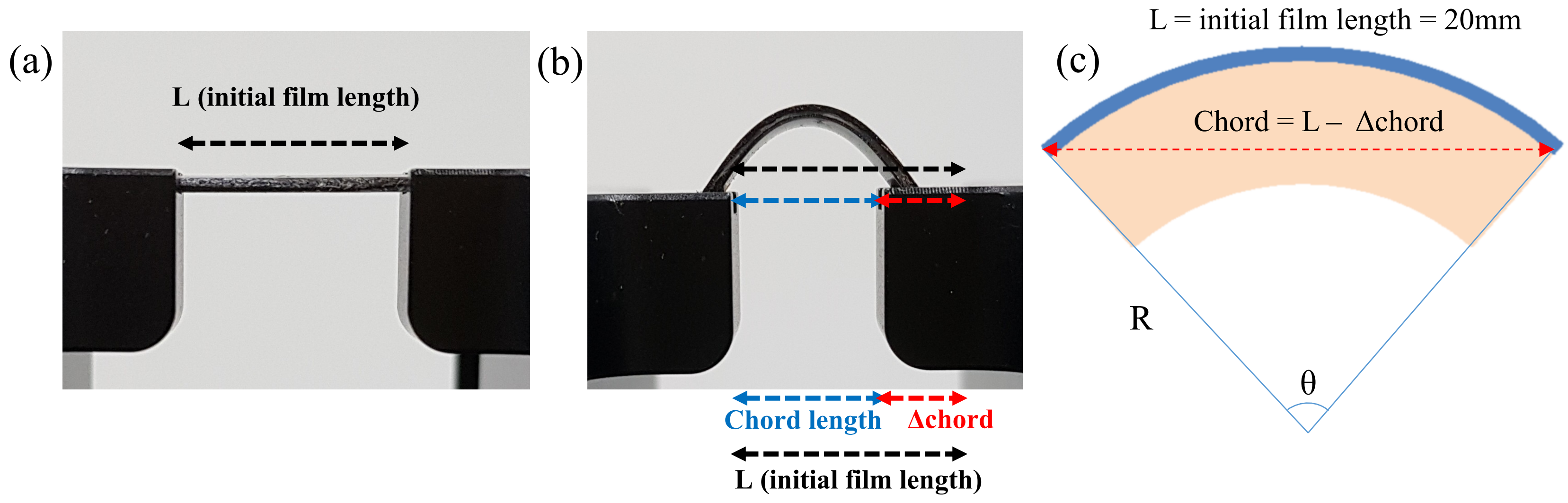


**Strain on Film**

$$\epsilon_{top} = (d_f + d_s)/2R \quad \text{....... eq (1)}$$

$\epsilon_{top}$ - In-plane strain of top surface
$d_f$ - film thickness (0.2um)
$d_s$ - substrate thickness (500um)

**Geometry relation**

$$2R\sin\left(\frac{\theta}{2}\right) = L - \Delta chord\,, \qquad R\theta = L$$

$$\Rightarrow 2R\sin\left(\frac{L}{2R}\right) = L - \Delta chord \quad \text{........eq (2)}$$

Figure S7. Unstrained (a) and strained (b) rhomhohedral GeTe (200 nm) film on a polyimide substrate (500 µm). The schematic (c) shows the geometric relation between chord and delta chord. Chord length is the modified specimen length along the in-plane direction, causing strain on the substrate. As the chord length is reduced, the specimen becomes curved and uni-axial stress is applied. We define the reduced chord length as delta chord. In addition, in-plane tensile strain of the top surface can be described as Eq. (1). From the geometric relation defined by Eq. (2), we can calculate the curvature R from L and delta chord, which are already known values. As a result, the in-plane tensile strain can be obtained by substituting the R value in Eq. (1)
